# An Iranian man with increased thigh mass due to a hydatid cyst

**DOI:** 10.3205/dgkh000355

**Published:** 2020-08-20

**Authors:** Fatemeh Samiee-Rad, Ali Emami

**Affiliations:** 1 Department of Pathology, Qazvin University of Medical Sciences, Qazvin, Iran; 2 Student Research Committee, Qazvin University of Medical Sciences, Qazvin, Iran

**Keywords:** hydatid cyst, intramuscular, primary, thigh, echinococcosis

## Abstract

**Background:** Hydatid cyst is a zoonotic infection caused by *Echinococcosis granulosus*. Primary single-intramuscular hydatid disease is rare, even in endemic regions of the world. Here we report the case of exceptional thigh mass due to a hydatid cyst in an Iranian man.

**Case presentation:** An 86-year-old man, initially presented to Velayat teaching hospital surgery clinic in May 2017 with a single right-thigh mass, but physical examinations of other organs were unremarkable. Based on sonographic findings, the differential diagnosis was hydatid cyst. He underwent surgical resection of the cyst. Histopathological results confirmed the diagnosis. There was no evidence of recurrence of the lesion during the 23-month follow-up.

**Conclusion:** Increase thigh mass due to a hydatid cyst is a rare event. In endemic regions with the presence of hydatid cysts, especially physicians of surgical clinics have to consider differential diagnosis of hydatid cysts in unusual locations in case of such a lesion.

## Zusammenfassung

**Hintergrund:** Die Hydatidenzyste, eine zoonotische Infektion, wird durch *Echinococcosis granulosus* verursacht. Primäre einzelne intramuskuläre Hydatidenerkrankungen sind selbst in endemischen Regionen der Welt selten. Wir berichten über den Fall einer außergewöhnlichen Oberschenkelmasse aufgrund einer Hydatidenzyste bei einem iranischen Mann.

**Kasuistik:** Ein 86 Jahre alter Mann wurde im Mai 2017 der Velayat-Klinik für Chirurgie mit einer vergrößerten Masse am rechten Oberschenkel ohne andere Nebenbefunde vorgestellt. Sonographisch wurde die Differentialdiagnose einer Hydatidenzyste gestellt, die chirurgisch reseziert wurde. Histopathologisch wurde die Hydatidenzyste bestätigt. Während der 23-monatigen Nachbeobachtungszeit gab es keine Hinweise auf ein erneutes Auftreten der Läsion.

**Schlussfolgerung:** Eine durch eine Hydatidenzyste verursachte vergrößerte Oberschenkelmasse ist ein seltenes Ereignis. In endemischen Regionen mit Vorkommen von Hydatidenzysten müssen insbesondere Ärzte chirurgischer Kliniken bei einer derartigen Läsion an ungewöhnlichen Stellen Hydatidenzysten differentialdiagnostisch in Betracht ziehen.

## Introduction

Hydatid cyst, cystic echinococcosis (CE) or hydatid disease is a zoonotic infection caused by one type of tapeworm, types *Echinococcosis granulosus* [1], [2]. This disease can be transmitted to humans by the fecal-oral route. The most infected sources are contaminated water and vegetables [3]. 

Dogs are the main host and humans are the incidental hosts [2], [4]. When the larvae enter the body of the host, they migrates through vessels and enter the liver bed [5]. Its ranges is throughout Asia, Australia, southern Europe, Africa and the Middle East [6], [7].

Some occupations which work closely with animals have the highest risk of the infection, such as tanners, slaughterhouse workers, shepherds, butchers, stockbreeders and veterinarians [8]. CE is endemic in regions all over the world; e.g., some parts of Europe are endemic regions for hydatid cyst infection. The highest incidences of CE (4.5/100.000) were reported in Bulgaria [7]. On the other hand, in Austria from 2011 to 2014, the average incidence stabilized at a median of 9 new cases of CE per year [9]. CE infestations have been reported from all countries in the Middle East, but the alveolar type is less common in Iran, Turkey, Iraq, and Tunisia [10]. Human cases have been reported from different parts of Iran. The prevalence of CE in southern Iran, South Tehran, and Shiraz were 5.4%, 5.9% and 6.7% respectively [11], [12], [13], and in Turkey, Iraq, and Tunisia, the prevalence were 6.9%, 3.7% and 10.41%, respectively[14], [15], [16]. 

This infection affects the liver and lungs in most cases (more than 90%), but all organs such as kidney, spleen, brain and other soft tissue can be affected [17]. CE rarely affects the musculoskeletal system. Subcutaneous cyst incidence depends on whether the cyst is primary or secondary, but overall incidence is 2% [18]. Nails, hair and teeth do not form cysts [19]. 

Hydatid cysts may be asymptomatic in most cases, but there are variety of symptoms depending on the size, location, and complications. Cyst rupture and immunological reactions are the most frequent complications [3], [6], [20]. An intramuscular cystic lesion may resemble a soft-tissue tumor, so even the slightest misdiagnosis can change the course of treatment [21]. Cystic tuberculosis is one of the differential diagnoses of thigh cyst. Imaging studies, drainage, associated cytological evaluation and pathology can distinguish it [22]. Another possible diagnosis of such a thigh lesion is sciatic nerve hematoma, which is associated with weak knee flexion [23]. Further, cystic lymphangioma can occur in the thigh and is a multilocular cystic lesion [24]. 

The diagnosis of this disease may be coincidental, or the patient may refer with a mass in the specific organ. Based on the radiological appearance, cyst viability and contents, hydatid cysts are classified into five stages. An active disease with clear contents and “water-lily” sign in radiology indicate the first and second stages. The third stage is characterized by the integrity of the cyst being compromised by the host immune system or drugs. The fourth and fifth stages include an inactive cyst and thick, calcified cyst walls [25]. One of the methods which can help diagnose hydatid cyst is serology, but the final pre-operative diagnosis is done by imaging. Ultrasound discriminates between a cyst and a solid lesion, shows the number of cavities in the cyst and differentiates it from organs. 

For a muscle cyst, MRI is the best imaging procedure, as it can show the location of the cyst and its relation to other structures. MRI can detect hydatid cysts in all parts of the body [20], [26].

The most effective treatment of hydatid cyst is radical surgery. The most dangerous complication during surgery is cyst rupture, because it causes anaphylactic shock and dissemination throughout the body. Using 0.5% cetrimide, 15% hypertonic saline, 1% silver nitrate and sodium hypochlorite solutions can kill the daughter cysts and decrease the risk of anaphylactic reactions [19]. 

The hydatid cyst has a specific character, and a long incubation time from exposure to definite diagnosis. For a definite diagnosis of CE, in addition to a complete medical history and physical examination, travel history and contact with animals play an important role [27]. The present patient had no additional or helpful documentation to facilitate diagnosing hydatid cyst.

On the other hand, one of the rarest systems to be affected by hydatid cyst is the musculoskeletal system (about 0.5–4%). Most of the time, it is an incidental finding in lung or liver, or by imaging studies in endemic regions [17]. However, based on our literature review, the occurrence of a thigh mass as the initial clinical presentation of hydatid cyst is very rare. This is because the cysts have a high oxygen requirement in the tissue for growth, but muscles have a low oxygen content and are high in lactic acid, which inhibits the growth of cysts and muscle contraction [17], [21], [28], [29]. Of course, the proximal muscles of the lower limb have greater muscle mass and blood supply [30]. Therefore, this case of skeletal muscle hydatid cyst in a non-endemic region in an elderly male without any job associated contact or history of travel to endemic regions was quite novel. 

The current paper reports this case and an associated literature review.

## Case presentation

Prior to entering the study, informed consent was obtained from the patient. 

The patient was an 86-year-old man presenting with a 2-month history of mass increase in the anterior part of the right thigh,who wa sreferred to the surgical clinic of Velayat teaching hospital of Qazvin in 2017. In last few weeks prior to presenting at the hospital, the mass had increased in size and the patient was aware of it. It prompted the patient to seek out a doctor. He had ischemic heart disease (IHD) and hypertension. His history of medications included nitroglycerin, aspirin (80 mg) and methyldopa. He had no past surgical history and his family history of CE and other diseases was negative. He was a retired grocer living in Qazvin city, which is not endemic for hydatid cyst. The patient had no contact with dogs, sheep, or other animals. He had no recent travel history to an endemic region, and his job was not related to CE, so suspecting hydatid cyst in this patient was not obvious. 

In appearance, he was not ill and denied any weight loss in the previous six months. The patient’s physical examination revealed a soft, non-tender and mobile mass inside the quadriceps femoris muscle. There were no skin changes or inguinal lymphadenopathy in right thigh. His laboratory, hematology, biochemistry and serologic findings were normal and erythrocyte sedimentation rate (ESR) was 65 mm/h. 

The differential diagnoses were abscess, hematoma and benign tumors (such as myxoma, cystic lymphangioma, and giant epidermoid cyst).

**Figure 1:**
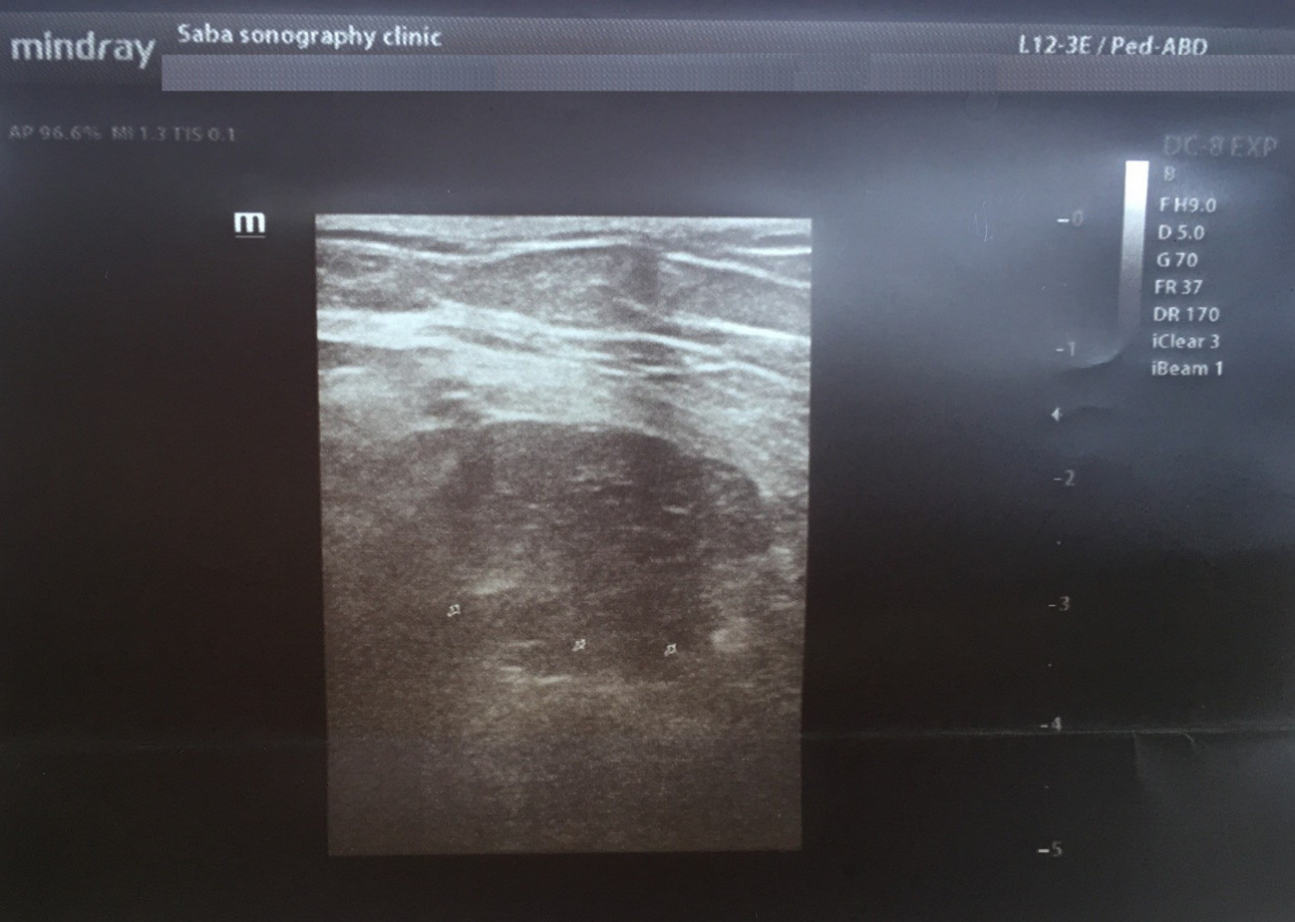
Ultrasound of the thigh revealed an encapsulated multi-cystic lesion with an indistinct margin (arrowheads) or a well-defined hyperechoic wall (arrow) corresponding to a calcified, non-vital hydatid cyst

Ultrasound findings demonstrated a hypoechoic encapsulated multi-cystic lesion (M: 9*8*6.5 cm) inside the anterior quadriceps femoris muscle. There was a smooth calcification in the cyst wall. The most important differential diagnosis based on imaging findings was hydatid cyst (Figure 1 (Fig. 1)).

**Figure 2:**
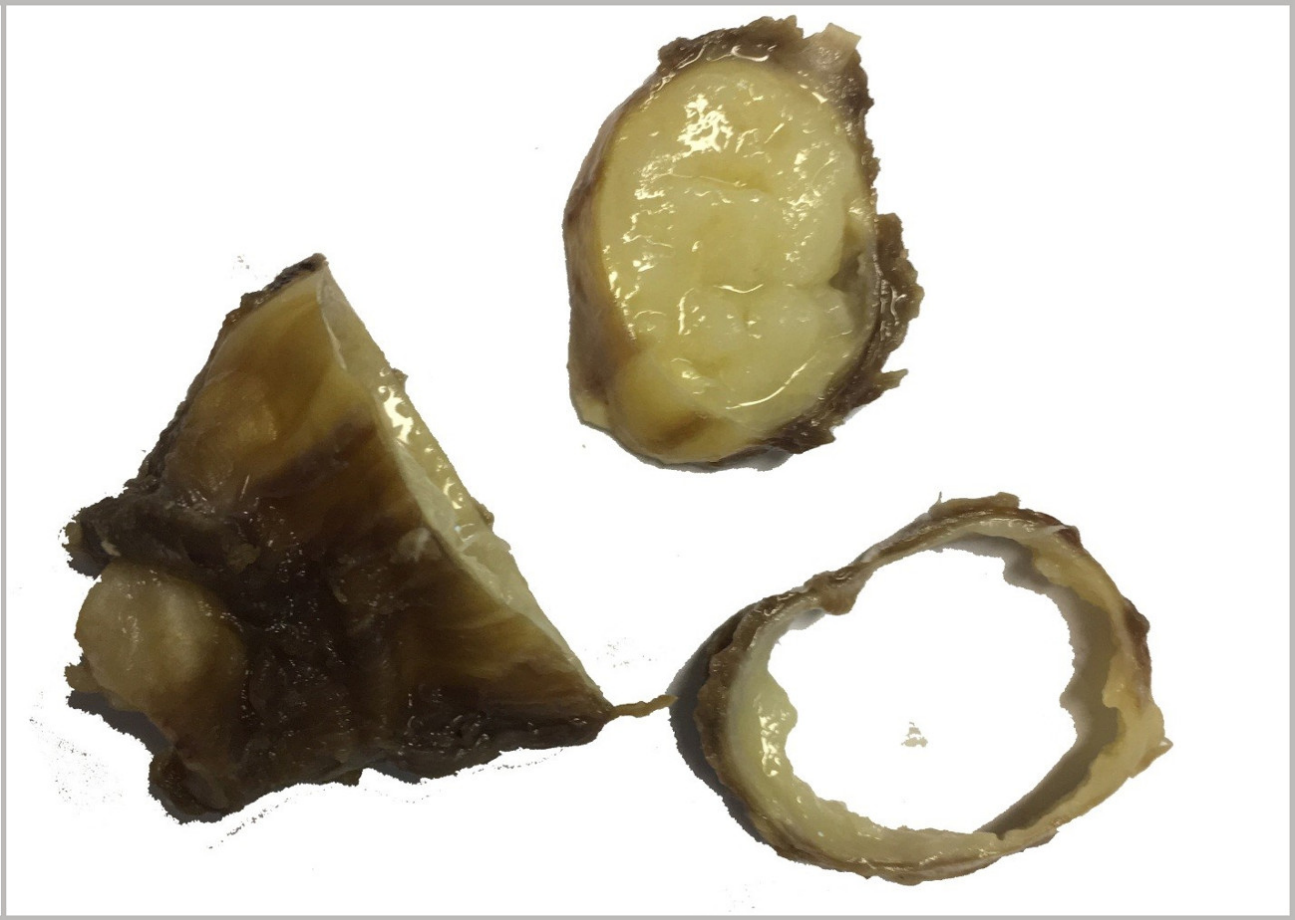
Postoperative view of the cystic mass, macroscopic sections; the cyst was filled with fragile particulate matter.

**Figure 3:**
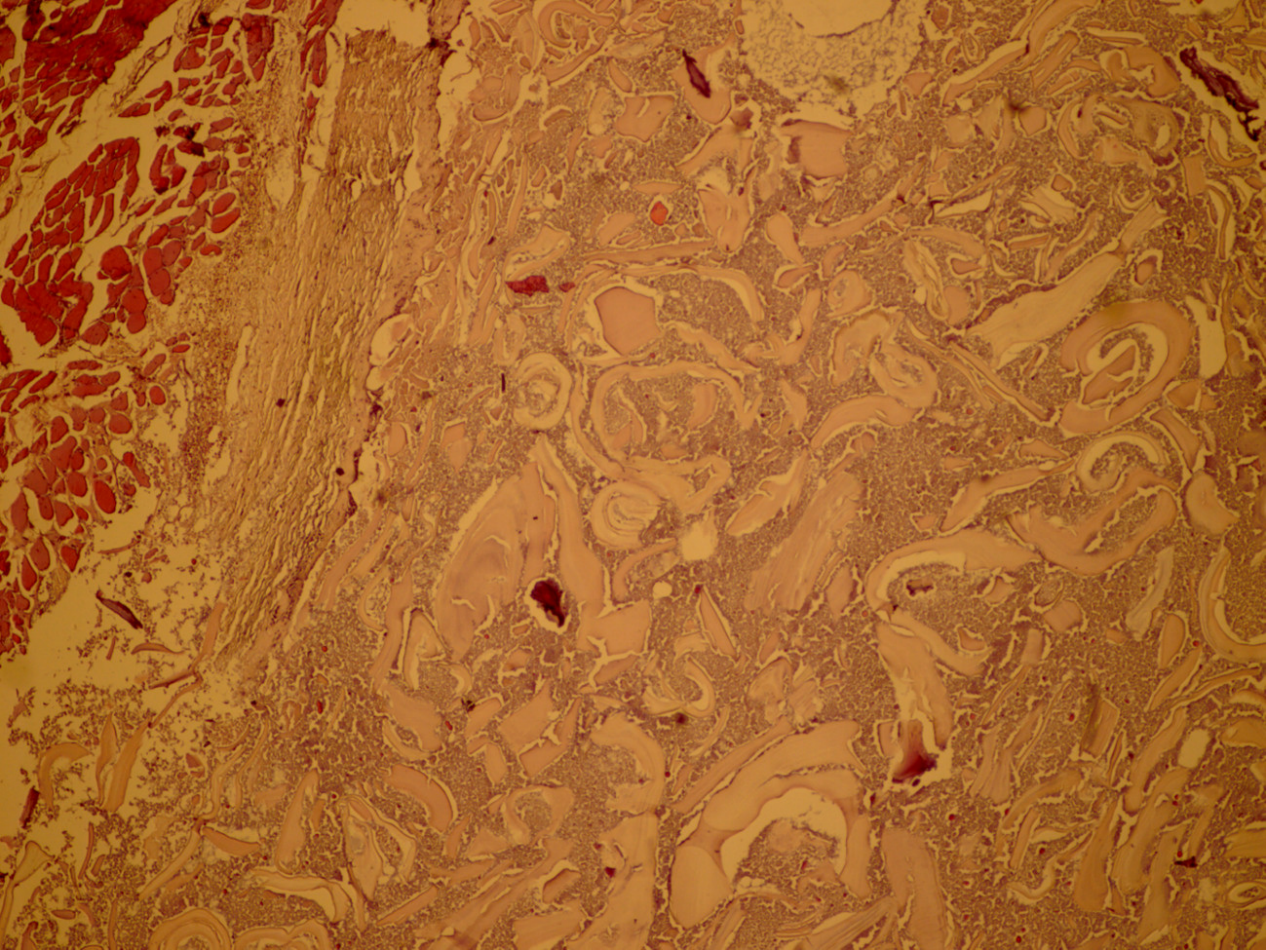
Thigh hydatid cyst from outside to inside revealed skeletal muscular bundles, a collagen capsule and a fragmented, acellular laminated layer, as well as protoscoleces admixed with necrotic material; 100×, Hematoxylin & Eosin.

**Figure 4:**
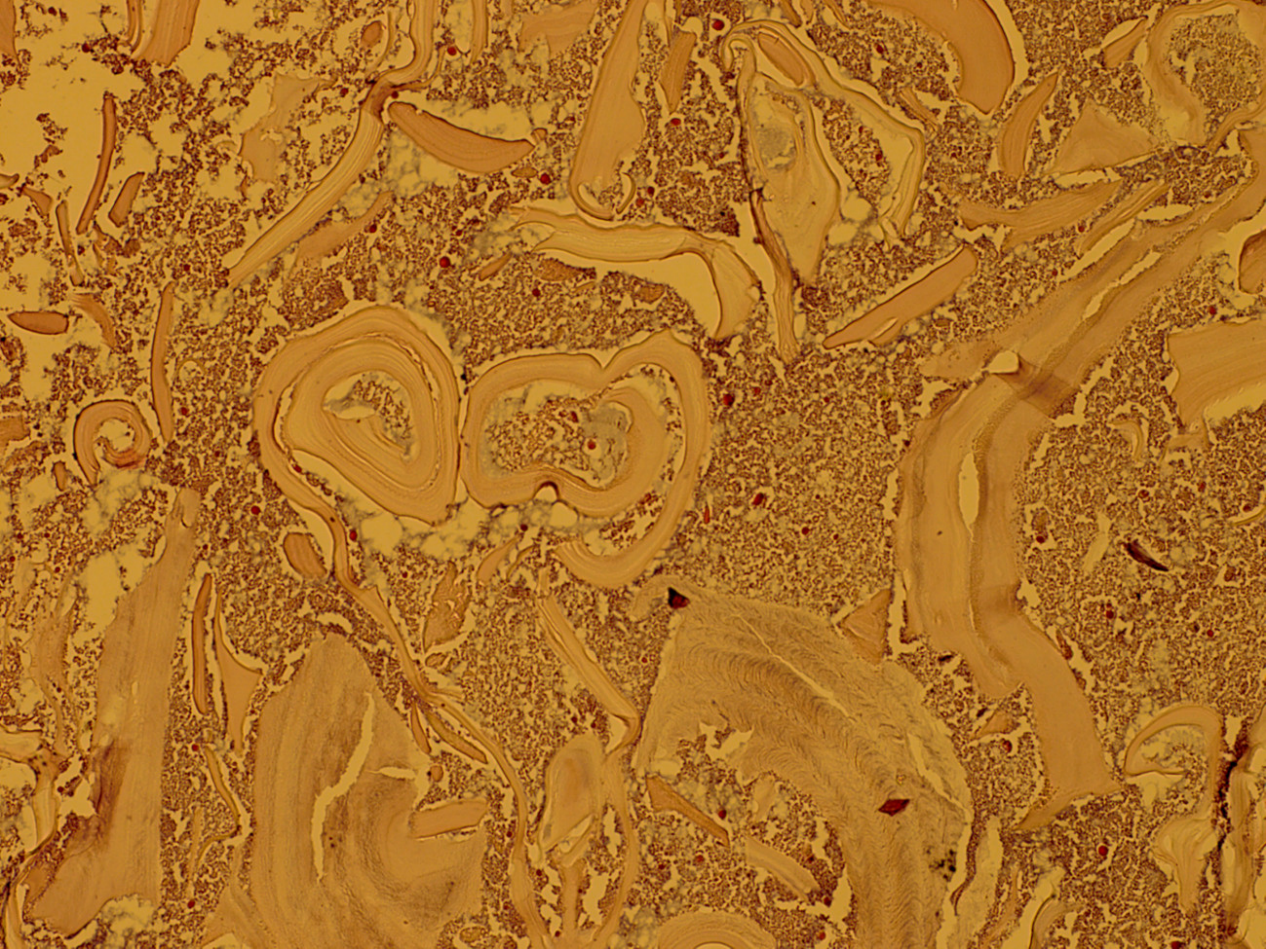
Thigh hydatid cyst revealed a fragmented, acellular laminated layer, admixed with necrotic material; 200×, Hematoxylin & Eosin.

**Figure 5:**
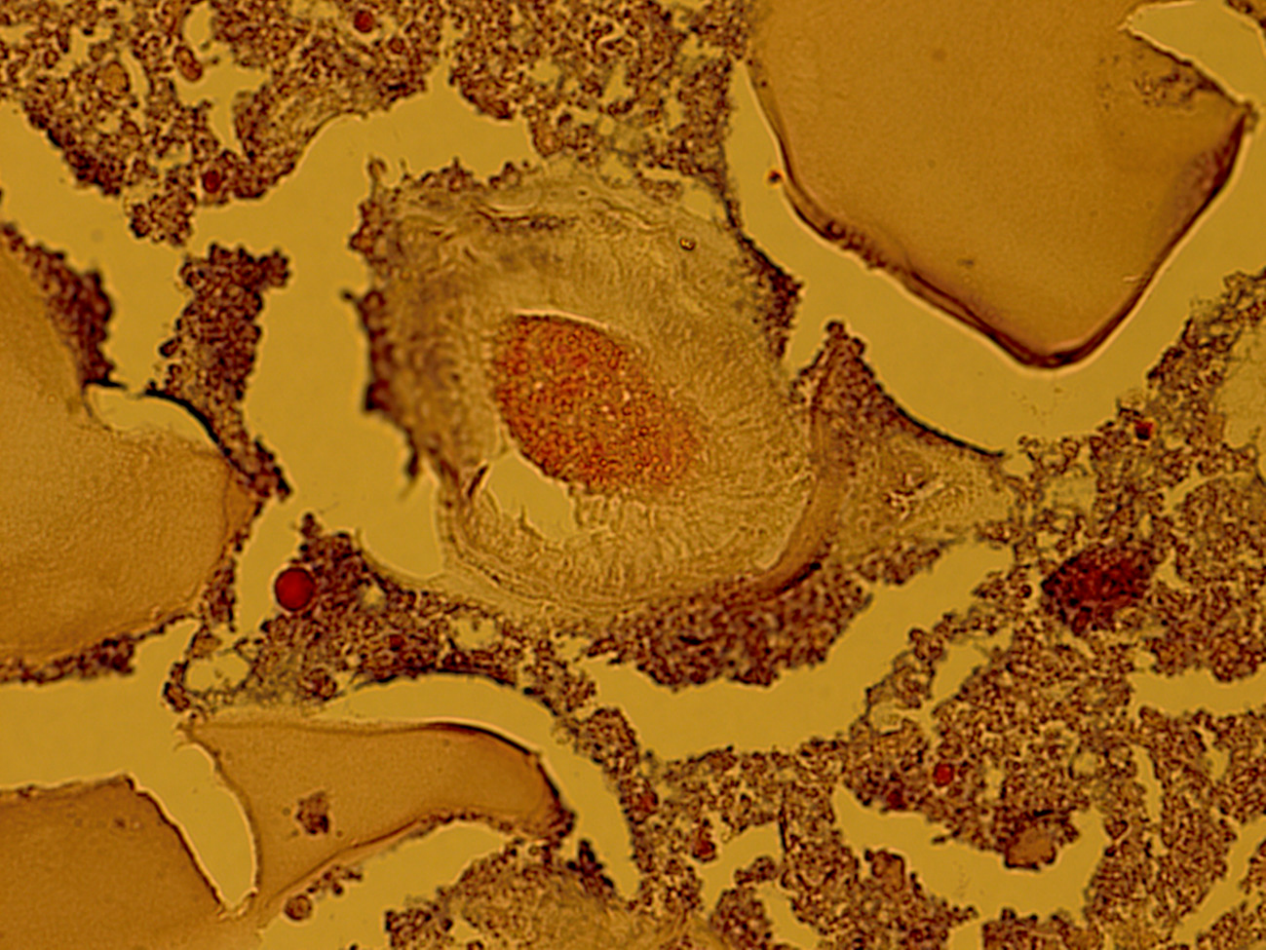
Thigh hydatid cyst revealed a fragmented, acellular laminated layer, protoscolex admixed with necrotic material; 400×, Hematoxylin & Eosin.

To exclude hydatid cyst in other organs, such as the liver and the lung, a pre-surgical CT scan of the chest and abdomen was performed, which chest and intra- abdominal organs were normal limit. Before surgery, the patient was prescribed albendazole. The patient that was suspected to have a hydatid cyst, underwent surgical resection of cyst. The cystic lesion was completely removed without damaging the cyst (Figure 2 (Fig. 2)). A review of the slides from resected cystic wall from exterior to interior showed skeletal muscular bundles, a collagen capsule containing a fragmented acellular laminated layer, and protoscoleces admixed with necrotic material (Figure 3 (Fig. 3), Figure 4 (Fig. 4), and Figure 5 (Fig. 5)).

The patient’s post-surgical course was non-remarkable. There was no evidence of recurrence of the lesion during the 23-month follow-up. The patient continued treatment with albendazole for three months. 

## Discussion

The imaging and histopathologic findings of our case are consistent with hydatid cyst referred with thigh mass. 

Echinococcosis is a larval infestation characterized by metacestode (hydatid) cysts in the intermediate host. This cyst consists of an inner germinal layer and an outer laminated layer. These layers are the parasite-derived layer, the interior of which is nucleated with daughter cysts, and brood capsules with scolices, while the outer part is hyalinized and acellular. The third layer is a host-produced fibrous capsule which is surrounded mainly by an inflammatory infiltration of lymphocytes, plasma cells, scant neutrophils leukocytes, eosinophils and occasional histiocytic aggregation; foreign body granulomatous reaction is also involved [31]. 

Sexual maturity of *E. granulosus* occurs in the small intestine within 4–5 weeks after the eggs enter via a gastrointestinal pathway. The larvae are released in the intestine and penetrate the epithelium to reach the lamina propria. The larvae spread in the body through the blood and lymph systems. Finally, in target organs, the larvae develop into a hydatid cyst [32]. Cytological findings of aspiration fluid may demonstrated clear fluid with particulate matter, which may contain scolices with hooklets and fragments of the laminated membrane [30].

Hydatid cysts occur in the lungs and liver or other sites of body but primary CE in the muscle of thigh is rare and reported in the literature [2], [3], [5], [17]. Because of their rich vascular supply, neck muscles are the other muscular part of the body in which hydatid cysts have been found [33]. Hydatid cyst is more likely to occur in the neck and trunk than at other locations in the musculoskeletal system, due to less muscle contraction and more vascularization of these areas [29]. CE of muscles presents with an enlarging, painful mass. Other complications can be nerve compression or allergic reactions due to rupture from trauma [34]. Our patient had a painlessly growing mass and no such complications.

In the case report by Tahir et al. [17], the patient had a painful mass on the inner aspect of right thigh. The MRI findings showed that the mass was related to the superficial femoral artery and the muscle. Our patient had no pain and his imaging reported no artery involvement. In another case report, a hydatid cyst was located in the adductor magnus muscle. The patient was a farmer living in a rural area of Iraq [35]. Salehi et al. [36] reported a biceps brachii CE in a butcher who had direct contact with animals (sheep and dog) and lived in Isfahan (an endemic location for CE in Iran).

Some clues can guide physicians to diagnosing this disease, e.g., occupation, travel history, occupation, and contact with animals and living in endemic locations. But diagnosing this specific disease in our patient without a related occupation or living in an endemic area was challenging.

The incidence of CE is not related to age, and occurs in all age groups. Case reports documented hydatid cysts in persons between 1 and 75 years of age [37]. Our patient was 86 years old.

Hydatid cyst manifests as a slow-growing mass resembling a soft-tissue tumor and can mimic symptoms of myositis or calcified hematoma [29], making diagnosis challenging. In addition to imaging (ultrasonography, CT and MRI), laboratory tests such as antibody and antigen detection can be used to find the definite diagnosis. Ultrasonography is inexpensive and easy to perform, but CT provides the best images for evaluating cyst dissemination [4]. Based on CT and ultrasound, other differential diagnoses of thigh mass, such as tumor, hematoma, tuberculosis and lymphangioma, were ruled out. Serological tests and the eosinophilia in blood count are not positive for all patients [38]. Our patient did not exhibit eosinophilia and his serological tests were negative. Eosinophilia occurs in the larval stage of echinococcosis. It has been reported that the body’s defense mechanism against the larvae consists of producing high levels of eosinophils, because the large size of the larvae prevent them from being phagocytized. The IgE-dependent mast cells localize eosinophils beside the parasite and the antiparasitic functions improve [31]. The larval stage was not present in our patient. 

Serological tests are available for this infection which help reach a definite diagnosis. The size, location and clinical stages of CE can affect the accuracy of serological tests. The same serological tests performed in different laboratories can change the sensitivities [39]. However, a negative serology result or CE cannot rule out the infection and in some cases, 30–40% of patients with hydatid cyst are antibody negative. The may be explained by the ability of this parasite to inhibit B cell activity and cellular proliferation.

The sensitivity of serological tests depends on the stage of disease; patients with inactive or early cystic stages have test sensitivity of 54.8%, but the sensitivity for patients with active cysts increases [27]. Our patient had an inactive cyst, i.e., CE stages 4 & 5.

Size and location of CE are important for complete healing. Perioperative albendazole treatment (at least four days before surgery) reduces the relapse rates, parasitic burden and risk of anaphylactic shock [40]. In our patient, resection surgery, albendazole therapy perioperatively and postoperatively eliminated the cyst and prevented the recurrence.

Because differential diagnosis and the radiological features were unclear, complete excision of the lesion was the best option for definite diagnosis [17]. Medication alone is not effective against hydatid cyst in the thigh, so surgery is the first step in treatment. Medication may be used preoperatively and postoperatively to decrease the recurrence rate [3]. 

Despite the exact and repeated review of the patient's history, some questions remained unanswered: How did the patient become infected with CE and what caused CE to infect his thigh?

## Conclusions

Thigh mass due to hydatid cyst is rare. Physicians in surgery referral treatment centers, especially those in endemic hydatid cyst areas, are much more likely to be familiar with and aware of this lesion in uncommon sites. Imaging and histopathological examinations help to accurately distinguish between hydatic cyst and other mass lesions of the thigh, and determine the outcome.

## Notes

### Competing interests

The authors declare that they have no competing interests.

### Acknowledgement 

We give our special thanks to the Clinical Research Center of Kosar Hospital and Mrs. Simindokht Molaverdikhani for assisting us in this project.
